# Improving growth and lipid accumulation in a novel oleaginous fungus *Fusarium solani* as a promising feedstock for biodiesel: optimization of growth medium and fatty acid characterization

**DOI:** 10.1186/s12866-026-05396-8

**Published:** 2026-07-21

**Authors:** Sedky H. A. Hassan, Mustafa A. Fawzy, Hana Sonbol, Albandary N. Alsaloom, Mostafa Koutb, Amany G. Ibrahim

**Affiliations:** 1 Department of Biology, College of Science, Sultan Qaboos University, Muscat, 123 Oman; 2 Botany and Microbiology Department, Faculty of Science, Assiut University, Assiut, 71516 Egypt; 3 Biology Department, College of Sciences, Taif University, P.O. Box 11099, Taif, 21944 Saudi Arabia; 4 Department of Biology, College of Science, Princess Nourah bint Abdulrahman University, P.O. Box 84428, Riyadh, 11671 Saudi Arabia; 5 Department of Biology, Faculty of Science, Umm Al-Qura University, Makkah, 24381 Saudi Arabia; 6 Botany Department, Faculty of Women for Arts, Science and Education, Ain Shams University, Cairo, 11566 Egypt

**Keywords:** *Fusarium solani*, Oleaginous fungi, Microbial lipid, Cultivation conditions, Biodiesel, Fuel characterization

## Abstract

**Background:**

The production of microbial lipids from oleaginous fungi is a promising method to support sustainable biofuels, but maximizing biomass and lipid accumulation requires carefully optimizing growth conditions using effective statistical approaches. The aim of this study was to enhance the biomass and lipid accumulation of a newly indigenous strain of the oleaginous fungus *Fusarium solani* OQ275245 by assessing the correlation between experimental results and independent design variables. The optimization process used a Box–Behnken experimental design with a response surface methodology, selecting starch, peptone, and inoculum size as independent parameters. The impacts of the parameters on the dependent responses (biomass yield, lipid yield, and lipid content) were investigated using response surfaces and contour plots, and the data was analyzed using ANOVA.

**Results:**

The optimal culture conditions were starch, peptone, and inoculum size at levels of 38.43 g/L, 0.53 g/L, and 1.0%, respectively. After validation of quadratic models, it was found that the optimized medium increased biomass yield by 1.06-fold, from 20.80 ± 1.90 g/L to 22.04 ± 2.51 g/L; lipid yield increased by 4.03-fold, from 1.31 ± 0.38 g/L to 5.28 ± 0.55 g/L; and the lipid content increased 3.81-fold, from 6.29 ± 1.36% to 23.96 ± 0.99% compared to the non-optimized medium. Hence, the use of RSM contributed to determining the concentrations of the studied variables to enhance biomass and lipid production in the fungal strain. Furthermore, fungal lipids analyzed by GC/MS were found to contain a high amount of long-chain saturated fatty acids, including myristic acid, palmitic acid, and stearic acid. This indicated that the *Fusarium* strain had a good fatty acid composition that can be effectively applied for production of biodiesel. Ultimately, we evaluated the characteristics of biodiesel generated by the *F. solani* strain, and the results met established criteria.

**Conclusions:**

These results suggest that *Fusarium solani* OQ275245 represents a sustainable and promising resource for the production of microbial lipids suitable for biofuel applications, and that the use of surface response methodology is an effective approach for improving the production of biomass and high-quality lipids.

**Graphical Abstract:**

**Figure:**
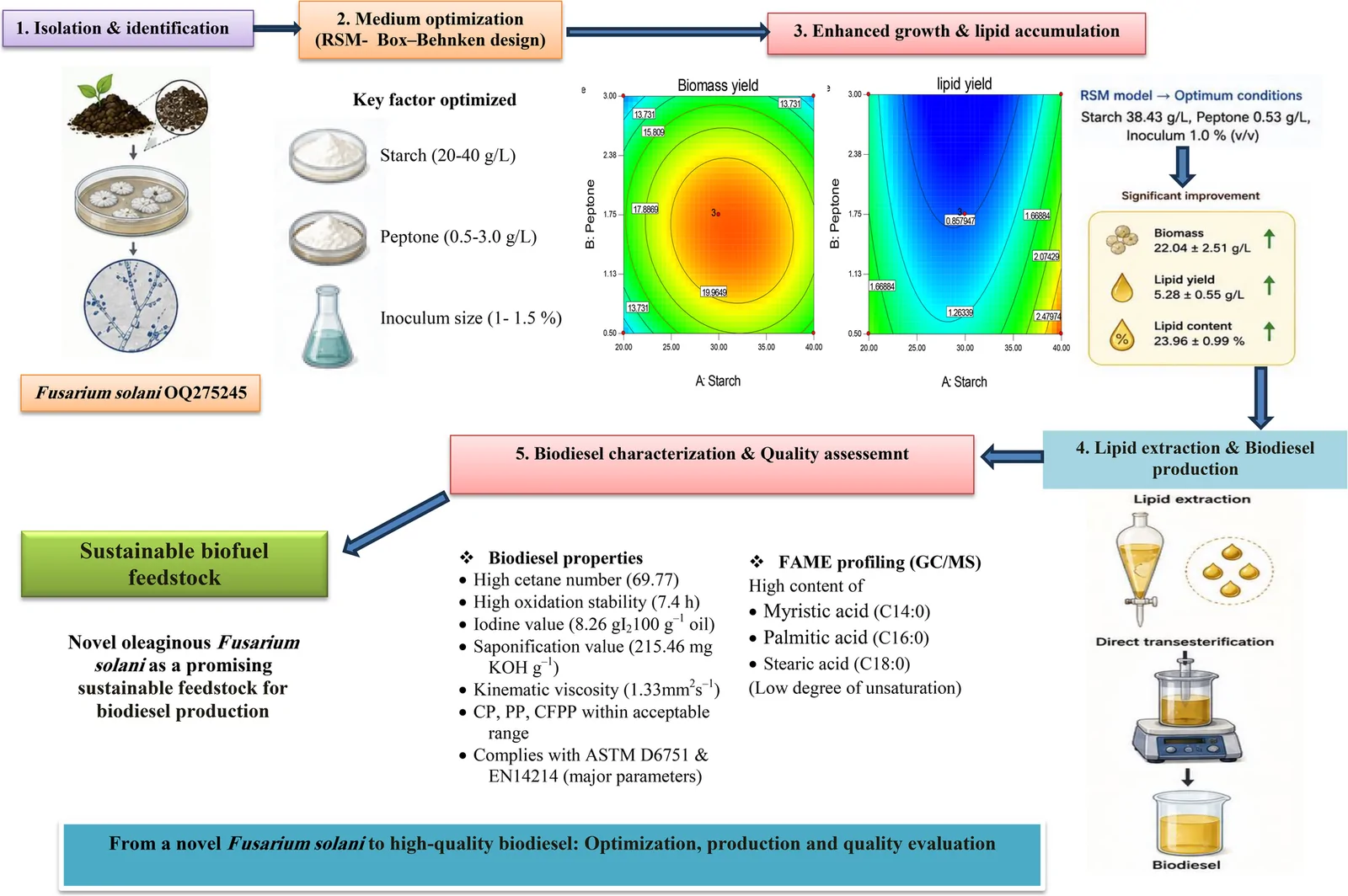

**Supplementary Information:**

The online version contains supplementary material available at https://doi.org/10.1186/s12866-026-05396-8.

## Background

Energy consumption has increased greatly in recent decades as a result of population development. In general the rise in energy demand has been met by the resources of fossil fuel, which are otherwise restricted and pose a major environmental risk [1]. As a result, there is an essential demand for alternative and sustainable fuels, such as biodiesel. Biodiesel (methyl or ethyl esters of fatty acid) is a leading candidate biofuel to replace petroleum-based diesel and is presently manufactured by transesterification of biomass triacylglycerol [2]. Biodiesel provides numerous environmental benefits, such as low greenhouse gas emissions, rapid biodegradation rate, high energy density, and lubricity [3]. Nevertheless, current biodiesel production depends upon traditional feedstocks, including plants and animals, causing competition for arable land, food security concerns, and high raw material costs [4]. On the other hand, biodiesel preparation from microbial groups such as yeasts, fungi, bacteria, and algae is gaining popularity due to the absence of these constraints and may provide an alternative to a biotechnology-based economy [5, 6].

It is known that oleaginous microorganisms accumulate lipids in the form of triacylglycerols in their cells or mycelia in proportions exceeding 20% w/w of their dry weight [7]. The possibility of using filamentous fungi for biodiesel production is currently supported by many researchers because of their capacity to grow on a variety of inexpensive feedstocks, low light energy requirements, capacity to produce about 70% lipids under stress, and ease of harvesting compared to yeasts, bacteria, and algae [8]. Identifying fungal species that can produce high lipid content to improve the economics of the process will be very helpful. *Aspergillus carneus* [9], *Mortierella isabellina* [10], *Mucor circinelloides* [11], *Mucor plumbeus* [12], *Fusarium solani*, and *F. oxysporum* [13, 14] are oleaginous filamentous fungi that can be used as biological templates for the production of biodiesel.

The cultivation method is crucial for efficient lipid production from oleaginous fungi in order to maximize industrial biofuel production by optimizing the growth process and producing useful byproducts. Some fungal species can produce large amount of lipids under variable growth circumstances, such as carbon and nitrogen concentrations, inoculation size, pH, incubation time, temperature, etc [15, 16]. Fungal cell proliferation and lipid accumulation are typically the two phases of intracellular lipid accumulation [10]. Fungi grow and multiply rapidly throughout the process of cell proliferation [15]. During this phase, available nutrients in the medium, mainly carbon sources, are rapidly consumed by fungal cells. The main goals of metabolic processes are biomass growth and cell division. As the process continues, the circumstances change in the direction of lipid accumulation. In this phase, the carbon supply in the growth medium is sufficient, but the nitrogen supply is depleted, causing cells to largely cease proliferation and switch metabolic activity to lipid synthesis [17]. Using certain fatty acid production pathways, fungi convert excess carbon into lipids. The availability of acetyl-CoA, which comes from carbon metabolism, helps in the transition to lipid production [18]. The lipids produced are stored as triacylglycerols, which provide energy to the fungi.

The amount and sources of carbon and nitrogen in the growth medium have a major impact on biomass production and lipid accumulation by oleaginous fungi [19]. Dey et al. [20] used organic (peptone) and inorganic [KNO_3_, NH_4_NO_3_, and (NH_4_)_2_SO_4_] nitrogen sources to assess the biomass and lipid productivity of *Alternaria* sp. and *Colletotrichum* sp. They found that both biomass and lipid yields were dependent on the nitrogen source, with peptone achieving maximum yields. Peptone stimulates enzymes in fungal cells responsible for lipid production because it contains various amino acids and other nutrients. The fatty acid profile and lipid accumulation by fungal species can also be influenced by the size and density of the inoculum [21]. Therefore, optimization of growth conditions and nutrient variables during the cultivation process is essential to enhance biomass and lipid accumulation by oleaginous fungi for biodiesel production [22]. Because the fatty acid profile of some oils can be considered as a gauge of fuel quality, the qualities of individual fatty esters in biodiesel have a significant impact on its characteristics.

Despite the increasing interest in oleaginous fungi for the production of lipids and biodiesel, *Fusarium solani* remains among the least studied fungal species in this field. Moreover, optimizing microbial lipid production remains challenging because lipid accumulation and fatty acid composition are highly strain-specific and strongly influenced by culture medium composition. In this regard, the current work examines a new indigenous *Fusarium solani* isolate obtained from the Jeddah region of Saudi Arabia as a potential feedstock for production biodiesel. The importance of this investigation lies in the assessing lipid-producing potential of this newly isolated strain and evaluating the quality of the biodiesel produced from it. As a result, the strain was molecularly identified and its growth and lipid production were improved by optimizing culture conditions, including the impacts of peptone, starch, and inoculum concentration, using response surface methodology (RSM). Furthermore, the physicochemical characteristics of the generated biodiesel were thoroughly assessed to demonstrate its suitability as a renewable energy source, supporting the development of sustainable biofuels, and providing insightful information for commercial and industrial applications.

## Methods

### Collection of samples and isolation of fungi

Six soil samples collected from different locations in the Second Industrial City of Jeddah, Saudi Arabia, were used to isolate the fungus strain. A serial soil dilution technique was used to isolate the fungi and then cultured on basal medium according to Mendes et al. [23] and kept on agar slants at 4 °C. Seven fungal isolates were obtained from the collected soil samples, purified and morphologically identified. One of these isolates was selected for further study based on the previously reported oleaginous potential of *Fusarium* species [13, 24]. The selected isolate was subsequently identified as *Fusarium solani* through molecular analysis and used for lipid production studies.

### Cultivation of fungal strain

The fungal strain used in this investigation was cultivated by inoculating 100 ml of basal medium (KH_2_PO_4_, 0.4 g L^–1^; MgSO_4_, 0.2 g L^–1^; NaCl, 0.4 g L^–1^; ZnSO_4_.7H_2_O, 0.001 g L^–1^; peptone, 3 g L^–1^; starch, 40 g L^–1^) in a 250 mL shake flask with 5 mL of autoclaved saline solution containing a spore suspension (1.5 ml; 1 × 10^6^). The pH was set to 6 ± 1 and the cultivation process was carried out in duplicate. The flasks were incubated for 7 days at 100 rpm and 30 °C.

### Fungal identification

The fungal isolate was molecularly identified based on amplification and sequencing of the large subunit ribosomal RNA gene (28 S rRNA). Fungal biomass was prepared under sterile conditions, followed by genomic DNA extraction using a standard protocol. PCR amplification was performed using the universal primers LR0R (5′-ACCCGCTGAACTTAAGC-3′) and LR5 (5′-TCCTGAGGGAAACTTCG-3′), targeting the 28 S rRNA region. The primer sequences and PCR conditions were adopted from previously published studies [25, 26].

The PCR products were verified by agarose gel electrophoresis, purified, and subjected to sequencing. The obtained sequences were compared with reference sequences available in the GenBank database using BLAST analysis.

Multiple sequence alignment was performed, and phylogenetic analysis was conducted using the Neighbor-Joining method implemented in MEGA 12. Bootstrap analysis was carried out with 1000 replicates to assess the robustness of the tree topology.

### Statistical optimization

Response Surface Methodology (RSM)-based Box–Behnken design (BBD) was used to optimize independent variables affecting biomass yield, lipid yield, and lipid content of the fungal strain. Design Expert, trial version 7 (State-Ease Inc. Minneapolis, USA), was used to design optimization experiments and perform statistical analysis. In order to determine the ideal parameter level for the indigenous fungal strain, this study employed three factors with three levels: carbon source (starch 20–40 g L–1), nitrogen source (peptone 0.5–3 g L–1), and inoculum size (1–1.5%), and 15 runs were used in the experimental design (Table 1). Growth medium was prepared using the basal medium described in the preceding section. A definite quantity of autoclaved saline solution containing Fusarium solani spores was added to 100 mL of culture media containing varying levels of the various variables obtained from BBD (Table 1), and the mixture was incubated for seven days.

**Table 1:** Experimental design and results for response surface analysis

Experiment no.	Factors (independent variables)			Responses (dependent variables)		
	A: Starch (g/L)	B: Peptone (g/L)	C: Inoculum size (%)	Biomass yield (g/L)	Lipid yield (g/L)	Lipid content (%)
1	20(− 1)	0.5(− 1)	1.25(0)	12.6 ± 2.55	2.34 ± 0.16	18.60 ± 0.70
2	40(+ 1)	0.5(− 1)	1.25(0)	14.6 ± 0.70	2.90 ± 0.21	19.89 ± 1.25
3	20(− 1)	3.0(+ 1)	1.25(0)	9.8 ± 3.10	1.45 ± 0.17	14.78 ± 3.10
4	40(+ 1)	3.0(+ 1)	1.25(0)	8.0 ± 0.04	1.36 ± 0.02	17.04 ± 0.30
5	20(− 1)	1.75(0)	1.00(− 1)	18.0 ± 2.10	2.99 ± 0.20	16.59 ± 1.09
6	40(+ 1)	1.75(0)	1.00(− 1)	18.0 ± 0.20	2.86 ± 0.04	15.88 ± 0.07
7	20(− 1)	1.75(0)	1.50(+ 1)	7.2 ± 0.06	1.42 ± 0.15	19.68 ± 0.49
8	40(+ 1)	1.75(0)	1.50(+ 1)	17.2 ± 2.20	3.11 ± 0.45	18.09 ± 3.70
9	30(0)	0.5(− 1)	1.00(− 1)	19.8 ± 0.85	2.61 ± 0.17	13.18 ± 2.42
10	30(0)	3.0(+ 1)	1.00(− 1)	14.0 ± 2.80	1.07 ± 0.04	7.67 ± 1.58
11	30(0)	0.5(− 1)	1.50(+ 1)	10.0 ± 3.31	1.13 ± 0.06	11.35 ± 0.49
12	30(0)	3.0(+ 1)	1.50(+ 1)	14.4 ± 2.50	1.47 ± 0.22	10.24 ± 1.10
13	30(0)	1.75(0)	1.25(0)	23.4 ± 2.30	1.05 ± 0.03	4.47 ± 0.35
14	30(0)	1.75(0)	1.25(0)	21.0 ± 1.12	0.80 ± 0.02	3.79 ± 0.63
15	30(0)	1.75(0)	1.25(0)	21.2 ± 1.30	0.62 ± 0.04	2.94 ± 0.16

Data are presented as mean ± standard deviation of three experiments

Data from the optimization experiments were fitted to a second polynomial equation as follows:

1 $$\begin{aligned}\mathrm{Y}&=\beta_{0}+\beta_{1}\mathrm{X}_{1}+\beta_{2}\mathrm{X}_{2}+\beta_{3}\mathrm{X}_{3}\\&+\beta_{12}\mathrm{X}_{1}\mathrm{X}_{2}+\beta_{13}\mathrm{X}_{1}\mathrm{X}_{3}+\beta_{23}\mathrm{X}_{2}\mathrm{X}_{3}\\&+\beta_{11}\mathrm{X}^{2}{_{1}}+\beta_{22}\mathrm{X}^{2}{_{2}}+\beta_{33}\mathrm{X}^{2}{_{3}}\end{aligned}$$

where Y represents the dependent response; β_0_ is the intercept; X_1_, X_1_, and X_3_ are the independent parameters of starch, peptone, and inoculum size, respectively; β_1_, β_2_ and β_3_ represent the linear coefficients; β_12_, β_13_ and β_23_ are the interaction coefficients between the three independent parameters; and β_11_, β_22_ and β_33_ are the quadratic coefficients, respectively.

### Verification of the predicted optimized conditions

The optimization was verified under optimal conditions: starch concentration of 38.43 g/L, peptone concentration of 0.53 g/L, and inoculum size of 1.0%, predicted by the developed model. The accuracy and applicability of the model were evaluated by comparing the actual and predicted values.

### Analytical methods

#### Biomass and lipid analysis

Fungal mycelia were filtered from the culture medium using Whatman grade 1 filter paper at the end of the cultivation period to measure biomass yield. Residual medium was then eliminated by washing the fungal filtrate with distilled water. Fungal mycelia were dried at 50–60 °C, and the biomass yield of the fungal strain was measured using gravimetric analysis. Biomass yield was given as grams of dry weight per liter of medium (g L^–1^).

The lipid content of the fungal strain was extracted using methanol–chloroform (2:1, v/v), and the extracted lipids were evaluated by sulfo-phospho-vanillin (SPV) approach according to Mishra et al. [27]. Briefly, 0.1 ml of extracted lipids was placed in a glass tube, 3 mL of H_2_SO_4_ was added, and the mixture was heated in a boiling water bath for 10 min. After cooling, 0.05 ml of hydrolyaste and 1.5 ml of SPV reagent were mixed and incubated at 37 °C for 10 min. The absorbance was then measured spectrophotometrically at 530 nm. Lipid yield was reported in grams of lipid per liter of medium, and lipid content was expressed as a percentage of dry weight.

### Transesterification and fatty acid methyl esters (FAME) analysis

The composition of FAMEs was assessed after transforming lipids from fungal biomass into methyl esters by direct transesterification for the fungal strain biomass, as described by Fawzy and Alharthi [28].

Fungal biomass was dried at 50 °C, and 0.5 g of the dried biomass was added to a 40 ml of mixture of methanol, chloroform, and concentrated hydrochloric acid (2:1:1). The mixture was then shaken at 120 rpm and left to stand overnight at 60 °C. FAMEs were then extracted by n-hexane and determined using a gas chromatograph/mass spectrometer (GC/MS) (Agilent 7890B with Agilent 5977 A detector) at a Central Laboratories Network, National Research Centre, Cairo, Egypt. The GC was equipped with a DB-5MS column (30 m × 0.25 mm internal diameter, 0.25 μm film thickness). Hydrogen was used as the carrier gas for the analyses, which were performed in splitless mode with a flow rate of 3.0 mL/min, an injection volume of 1 µL, and the following temperature program: 40 °C for one minute; increased at 10 °C/min to 200 °C and held for one minute; increased at 20 °C/min to 220 °C and held for one minute; and finally increased at 30 °C/min to 320 °C and held for ten minutes. The injector and detector temperatures were maintained at 250 °C and 320 °C, respectively. Electron ionization (EI) at 70 eV was used to obtain mass spectra, with a solvent delay of 1.3 min and a spectral range of m/z 50–600. The mass and Quadr temperatures were set at 230 °C and 150 °C, respectively. Identification of the various constituents was achieved by comparing their spectrum fragmentation patterns with data from the Wiley and NIST Mass Spectral Library.

### Determination of biodiesel characteristics

Biodiesel Analyzer© software version 2.2 [29] was used online to calculate various characteristics of fungal biodiesel based on the FAMEs composition. Cetane number (CN), saponification value (SV; mg KOH g^–1^), iodine value (IV; gI_2_100 g^–1^ oil), oxidation stability (OS; h), degree of unsaturation (DU; %), density (ρ; g cm^–3^), kinematic viscosity (kυ; mm^2^ s^–1^), high heating value (HHV; MJ kg^–1^), cold filter plugging point (CFPP; °C), cloud point (CP; °C), pour point (PP; °C), and long chain saturated factor (LCSF; %), were some of the biodiesel characteristics analyzed.

### Statistical analysis

Statistical analysis was carried out using analysis of variance (ANOVA). Statistical significance was defined at the 5% level as *p* < 0.05, and at the 10% level as 0.005 < *p* value < 0.1. All trials were performed in duplicate, and the results were analyzed to evaluate the regression coefficient of the experimental data, Fisher test (*F*-test), and the corresponding *p*-value (*F*). 3-D response surface and contour graphs for biomass yield (g L^–1^), lipid yield (g L^–1^), and lipid content (%) of the fungal strain were calculated and plotted based on the quadratic polynomial model by keeping one independent parameter at the center level, in order to assess the relationships between the various parameters.

## Results and discussion

### Identification of fungal isolate

**Fig. 1:**
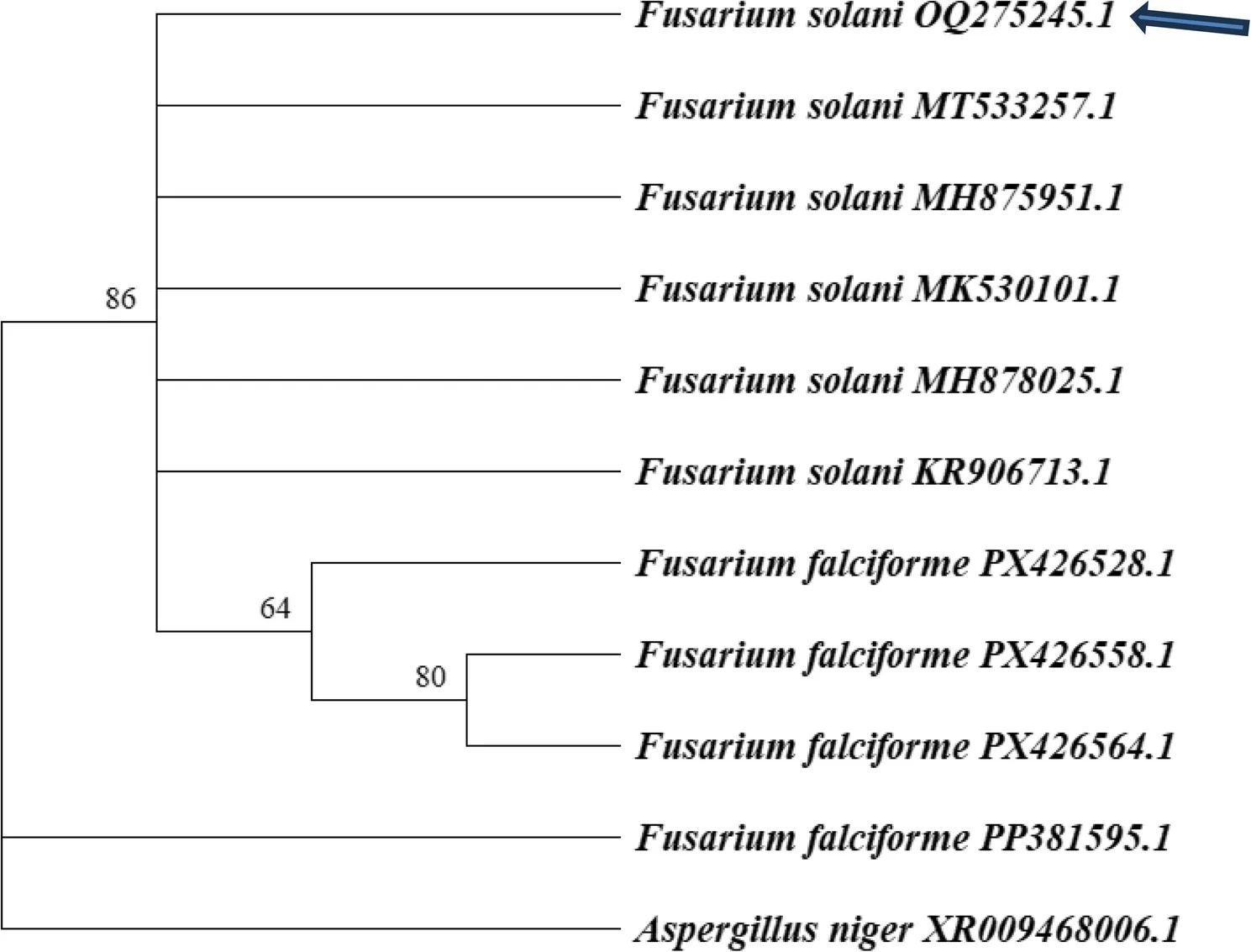
Phylogenetic tree based on partial 28 S rRNA gene sequences showing the relationship between the studied fungal isolate and related taxa from the GenBank database. The tree was constructed using the Neighbor-Joining method in MEGA 12 with 1000 bootstrap replicates. Bootstrap values (> 50%) are indicated at the nodes. The scale bar represents 0.02 substitutions per site

The molecular identification based on partial 28 S rRNA gene sequencing confirmed that the fungal isolate belongs to Fusarium solani. Phylogenetic analysis using the Neighbor-Joining method revealed that the studied isolate clustered within the Fusarium solani clade, closely related to reference strains retrieved from the GenBank database. The clustering showed strong statistical support with a bootstrap value of 86%. The phylogenetic tree (Fig. 1) was constructed with a scale bar representing 0.02 substitutions per site, reflecting the evolutionary distance among the analyzed sequences. The obtained sequence was deposited in the GenBank database under accession number OQ275245 (https://www.ncbi.nlm.nih.gov/nuccore/2428741467).

### Box–Behnken design for optimization of biomass yield, lipid yield and lipid content of *F. solani OQ275245*

The nature of the fungal species and growth conditions, such as temperature, pH, inoculum concentration, incubation duration, and nitrogen and carbon sources, influenced the amount of biomass and lipid accumulation [30]. In this work, the influence of interactions between the studied factors, including starch, peptone, and inoculum size, on biomass and lipid production by the oleaginous isolate *F. solani* OQ275245 was evaluated using a three-factor BBD within the RSM framework. Table 1 lists the entire experimental design with its values in actual and coded form, as well as the minimum and maximum ranges of the independent variables.

### Impact of independent parameters on the biomass yield of *F. solani* OQ275245

The Box–Behnken experimental design was used in order to ascertain the optimal circumstances for biomass production by F. solani OQ275245 after a 7-day incubation period. According to Table 1, run 13 produced the highest biomass yield (23.4 g/L) under fungal cultivation conditions containing soluble starch (30 g/L), peptone (1.75 g/L), and inoculum size (1.25%), at a fixed temperature of 20 °C and pH 6.

Regression coefficients and statistical analysis using ANOVA and response surface graphs were determined based on the data of the Box–Behnken design. The ANOVA data, which assesses the quadratic model’s significance, are displayed in Table 2. The data revealed that the F-value of the predicted model was 7.70 (p = 0.018), indicating that it was significant. In comparison to the pure error, the F-value of the lack of fit was 0.212, displayed non-significant behavior. This could be revealed by the relationship between the actual and expected data, as indicated in Fig. S1, which shows that the yield of fungal biomass is accurately expected by the polynomial quadratic model.

**Table 2:** ANOVA analysis of quadratic model for biomass yield

Source	Coefficient estimate	Sum of Squares	Degrees of freedom	Mean Square	F-value	*p*-value
Model		334.7	9	37.19	7.70	0.018
*A*-Starch	1.28	13.00	1	13.01	2.69	0.162
*B*-Peptone	-1.35	14.58	1	14.58	3.02	0.143
*C*-Inoculum size	-2.63	55.13	1	55.13	11.41	0.019
*AB*	-0.95	3.61	1	3.61	0.75	0.427
*AC*	2.50	25.00	1	25.00	5.17	0.072
*BC*	2.55	26.01	1	26.01	5.38	0.068
*A²*	-5.03	93.45	1	93.54	19.36	0.007
*B²*	-5.58	115.10	1	115.10	23.82	0.004
*C* ^*2*^	-1.73	11.09	1	11.09	2.30	0.190
Residual		24.16	5	4.83		
Lack of Fit		20.61	3	6.87	3.87	0.212
Pure Error		3.55	2	1.77		
Cor. Total		358.86	14			

Standard Deviation = 2.19, Mean = 15.28, C.V.%= 14.39, R²= 0.933, Adjusted R²= 0.812, Predicted R²= 0.688, Adequate Precision = 7.34

The high determination coefficient (R^2^= 0.933) and adjusted R^2^ (0.812) showed that the model had good fit and predictive capacity [31]. There was a good correlation between the experimental and expected data, as evidenced by the fact that adjusted R^2^ was in acceptable agreement with the predicted R^2^ [32]. Simultaneously, the model demonstrated good accuracy and dependability of the experiments, as indicated by the coefficient of variation (CV) value of 14.39%. Additionally, the signal was considered appropriate based on the adequate precision ratio of 7.34, as a signal-to-noise ratio higher than 4 is desirable [33]. According to these findings, the model employed in this investigation can be applied to optimize the growth circumstances of *F. solani* OQ275245, because the model allowed prediction of how different parameters affect the biomass production of *F. solani*. The significance of each coefficient, which can reveal the pattern of the relationships between the independent parameters, was examined using *p*-values derived from ANOVA analysis (Table 2), and the corresponding parameter is significant when the *p*-level is small (*p*˂ 0.05).

Table 2 showed that none of the factor interactions had a significant impact on the biomass yield of the fungal strain, and only the linear term of inoculum size (C) and the quadratic effects of starch (A^2^) and peptone (B^2^) were statistically significant.

The following equation was obtained by fitting a polynomial model for biomass yield (Y, g/L) according to the experimental data:

2 $$\begin{aligned}\mathrm{Y}_\text{Biomass yield}&=21.86+1.28\:\mathrm{A}-1.35\:\mathrm{B}-2.63\:\mathrm{C}\\&-0.95\:\mathrm{AB}+2.5\:\mathrm{AC}+2.55\:\mathrm{BC}\\&-5.03\:\mathrm{A}^{2}-5.58\:\mathrm{B}^{2}-1.73\:\mathrm{C}^{2}\end{aligned}$$

where A, B and C are the coded terms of starch, peptone and inoculum size, respectively. Positive values of the independent parameters demonstrate that the response increases as the parameter’s level increases and vice versa for the negative parameter values.

**Fig. 2:**
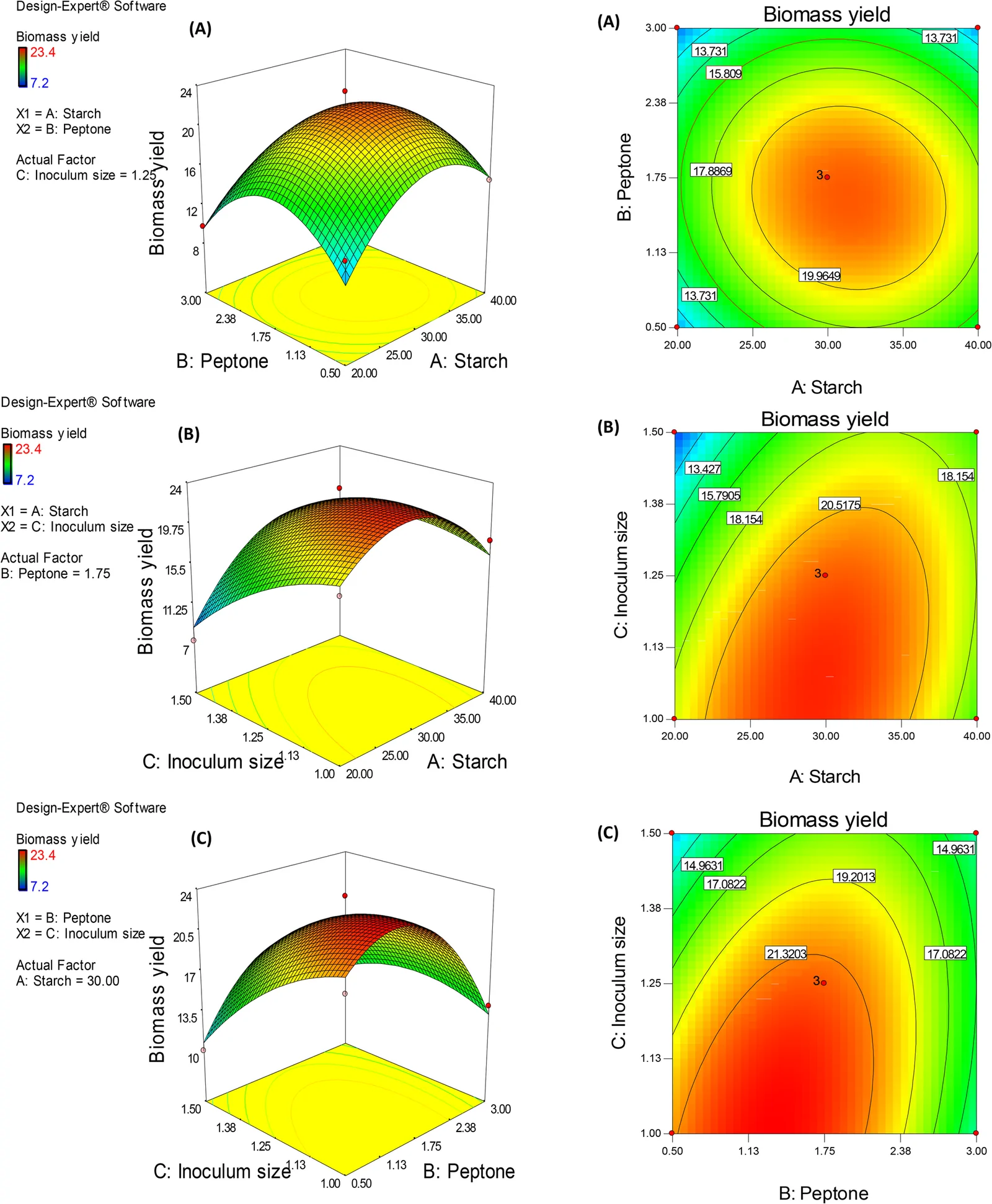
Three dimensional (3D) response surface and contour plots showing interactive effect of independent variables: (**A**) starch and peptone, (**B**) starch and inoculum size, and (**C**) peptone and inoculum size, on biomass yield modeled by Box–Behnken design

The interactive impact of the variables on the biomass production of the *F. solani* strain was visualized using a 3-D surface graph and contour plots, and the results are presented in Fig. 2. Plots of the relationships between starch and peptone concentrations and biomass yield showed that the optimal concentrations of starch and peptone are 30 g/L and 1.75 g/L, respectively (Fig. 2A). These findings are consistent with the study by Xia et al. [34], who reported that the progressive rise in *Mucor circinelloides*’ biomass and glucose consumption coincided with the increase in nitrogen concentration in the medium. According to Hashem et al. [35], peptone produced the maximum biomass (12.44 g/L) of *Syncephalastrum racemosum* and was the best source of nitrogen for cell growth.

As seen in the plot in Fig. 2B, the biomass production of the fungal strain increased with increasing starch level and decreasing inoculum size. The biomass peaked at 20.51 g/L at a starch concentration of 30 g/L, indicating that the soluble starch concentration met the strain’s growth requirements. ANOVA analysis supported these findings, showing that a high and positive value of the coefficient estimate of starch in the linear term (1.28; Table 2) indicates a positive and statistically insignificant impact of starch on *F. solani* biomass yield (*p* ˃ 0.05) and on enhanced fungal growth at increased the starch concentration (Fig. 2B). In this regard, Carvalho et al. [36] observed that high levels of starch as the sole carbon source for *Mucor circinelloides* URM4182 growth produced a high biomass production of 6.7 g/l with a lipid content of 31%.

Figure 2C showed the impact of peptone and inoculum size on the biomass yield of *F. solani* OQ275245. It can be noticed that the highest fungal growth (21.32 g/l) was achieved with a peptone of approximately 1.75 g/l and an inoculum size of ˂ 1.25%. High inoculum concentration may result in lower fungal growth because of the limited availability of medium constituents for *F. solani* to provide the highest biomass growth [37]. Conversely, fungal growth in reduced and dense inoculum pellets encourages growth and lipid accumulation because there is less limitation in the diffusion of oxygen and other nutrients through the growth medium. The impact of inoculum size on fungal growth was documented by Osman et al. [38], who found that an inoculum size of 5% for the oleaginous yeast *Rhodotorula diobovata* produced the highest dry biomass (11.84 g/L), and an inoculum size of 30% produced the lowest biomass (9.54 g/l).

### Impact of independent parameters on the lipid yield of *F. solani* OQ275245

The quantity of lipids generated by *F. solani* OQ275245 changed greatly based on the type and level of the variables tested. From Table 1, it can be observed that the maximum lipid yield (3.11 g/l) was obtained in experiment 8 under the conditions of 40 g/L starch, 1.75 g/L peptone, and 1.50% inoculum size. In accordance with previous studies by Hashem et al. [35], *Syncephalastrum racemosum* produced the maximum quantity of lipid content and total lipid (23.14% and 2.88 g/L, respectively) after using the medium supplemented with peptone and starch.

Table 3 displays the ANOVA results for the polynomial quadratic model of lipid yield. The *p*-value of the quadratic model, lack-of-fit test, the sum of squares, and R^2^ value demonstrated that the quadratic polynomial equation was the best regression model for the experimental results, which is similar to the biomass yield response.

**Table 3:** ANOVA analysis of quadratic model for lipid yield

Source	Coefficient estimate	Sum of Squares	Degrees of freedom	Mean Square	F-value	*p*-value
Model		10.47	9	1.16	13.24	0.0055
*A*-Starch	0.26	0.52	1	0.52	5.93	0.0441
*B*-Peptone	-0.45	1.65	1	1.65	18.75	0.0075
*C*-Inoculum size	-0.30	0.71	1	0.71	8.14	0.0357
*AB*	-0.16	0.10	1	0.10	1.19	0.3256
*AC*	0.46	0.83	1	0.83	9.44	0.0277
*BC*	0.47	0.88	1	0.88	10.00	0.0250
*A²*	1.11	4.52	1	4.52	51.47	0.0008
*B* ^*2*^	0.09	0.03	1	0.03	0.31	0.6002
*C²*	0.66	1.63	1	1.63	18.58	0.0076
Residual		0.44	5	0.09		
Lack of Fit		0.35	3	0.12	2.58	0.2914
Pure Error		0.09	2	0.05		
Cor. Total		10.91	14			

Standard Deviation = 0.29, Mean = 1.81, C.V.%= 16.35, R²= 0.959, Adjusted R²= 0.887, Predicted R²= 0.694, Adequate Precision = 9.38

A comparison of the observed lipid production by F. solani with the expected production is shown in Fig. S2, which indicates a high degree of agreement between the actual and expected values. Thus, the obtained findings demonstrated the correctness and usefulness of the established model to investigate the effects of starch, peptone, and inoculum size on lipid production by Fusarium isolate. ANOVA analysis also showed that peptone had the major influence on the lipid yield of the *Fusarium* strain (*p* = 0.007), followed by inoculum size (*p* = 0.035), and starch concentration (*p* = 0.044; Table 3). The lipid yield of *F. solani* was also significantly increased by the combining starch and inoculum size with a *p* value of 0.027 and peptone and inoculum size (*p* = 0.025). Additionally, the lipid yield was significantly impacted by the quadratic factors of starch (*p*˂ 0.001) and inoculum size (*p*˂ 0.01).

The general form of the quadratic equation obtained for lipid yield is displayed below (3):

3 $$\begin{aligned}\mathrm{Y}_{\text{Lipid yield}}&=0.82+0.26\:\mathrm{A}-0.45\:\mathrm{B}-0.30\:\mathrm{C}\\&-0.16\:\mathrm{AB}+0.46\:\mathrm{AC}+0.47\:\mathrm{BC}\\&+1.11\:\mathrm{A}^{2}+0.09\:\mathrm{B}^{2}+0.66\:\mathrm{C}^{2}\end{aligned}$$

**Fig. 3:**
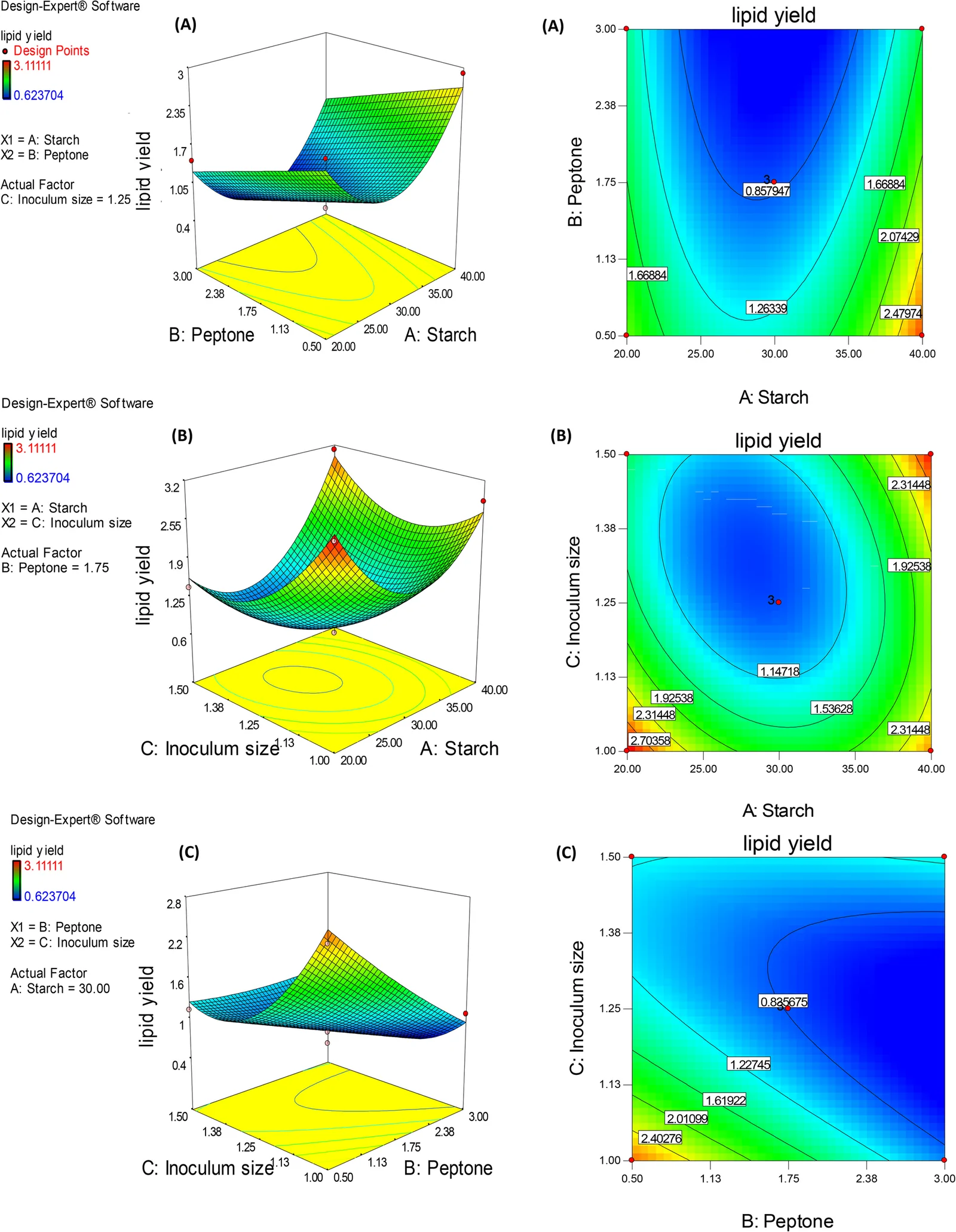
Three dimensional (3D) response surface and contour plots showing interactive effect of independent variables: (**A**) starch and peptone, (**B**) starch and inoculum size, and (**C**) peptone and inoculum size, on lipid yield modeled by Box–Behnken design

The mutual impacts of initial different starch and peptone concentrations on the lipid yield of the *F. solani* strain at a fixed inoculum concentration are depicted in Fig. 3A as three-dimensional and contour plots. The figure showed that higher starch concentration (40 g/L) improved the quantity of lipid yield compared to the same higher peptone concentration (3 g/L) with lesser starch. This interacting impact emphasizes the significance of optimizing both variables simultaneously. Although the effect of starch concentration alone was significant (*p* ˂ 0.05, Table 3), the interaction between starch and peptone was not statistically significant, suggesting that their influences were mostly independent within the examined range. In actuality, fungi synthesize cellular lipids when their nitrogen supply is insufficient and carbon supply is excessive [11]. Lipid production by lipid-producing strains is highly dependent on carbon sources, since these are closely linked to fungal growth and various metabolites. Fungi can grow and produce lipid-free biomass by using the carbon source present in the culture medium. However, when there is too much carbon in the growth medium, some of the carbon flux is exploited for lipid production [39]. On the other hand, stored intracellular lipid is released and used to support cell division and producing lipid-free biomass if the concentration of carbon in the culture medium is limited or when carbon supplies from extracellular sources are depleted [30].

The impacts of inoculum size and starch concentration on lipid yield of *F. solani* are shown in Fig. 3B. The results indicated that at lower starch levels, increasing inoculum size has a negative impact on lipid production; however, this effect is offset when starch concentrations reach 40 g/L. These findings showed that the lipid yield of *F. solani* is significantly impacted by the interaction between inoculum size and starch concentration, which was supported by ANOVA analysis, as previously reported. Inoculum size has a major influence on cellular lipid production by oleaginous fungi. According to Costa et al. [40], depletion of oxygen and nutrients in the growth medium may be the cause of the inhibitory influence on lipid production at larger inoculum sizes.

3-D and contour plots (Fig. 3C) of lipid production by *F. solani* OQ275245 at various peptone levels and inoculum size demonstrate an increase in lipid yield (2.40 g/L) under limited conditions of both parameters. Higher peptone concentrations and inoculum size independently had an inhibitory impact on lipid production by *F. solani*. This enhancement in lipid production is consistent with the observations of Venkata and Venkata [41], who reported that *Aspergillus awamori* accumulated more lipids when nitrogen content was reduced and reached the highest level when nitrogen was exhausted.

### Impact of independent parameters on the lipid content of *F. solani* OQ275245

The utilization of lipid-producing strains for lipid production requires strains with a high capacity for lipid accumulation. The fungal strain employed in this investigation has the ability to grow well for up to seven days in a nitrogen-limiting, carbon-rich medium. Table 1 indicates Box–Behnken design data, which indicate that the lipid content of *F. solani* OQ275245 varied between 2.94% and 19.89%. The maximum lipid content was recorded in run 2 (19.89%), using 40 g/L starch, 0.5 g/L peptone, and 1.25% inoculum size. In line with our findings, Hashem et al. [42] found that the optimal amount of soluble starch for *Mucor racemosus* MG547571 for lipid production is 40 g/L. Additionally, Longmatcha [43] found that *Aspergillus* sp. MMERU 24 had maximum lipid content (23.02 ± 0.47%) at a low peptone concentration of 1 g/L. Our findings are also consistent with those recorded by Sayeda et al. [44], who stated that *Fusarium oxysporum* NRC2017, employing an inoculum size of 1%, produced the highest amount of lipid (59.88%).

Table 4 shows the analysis of variance (ANOVA) for lipid content in *F. solani* OQ275245. As in the response of biomass yield and lipid production by *F. solani*, the polynomial regression model was the best-fitting model to the actual data. However, comparing the regression model of lipid content to biomass or lipid yield, the *F*-value of lipid content was the highest among all quadratic models (*p* = 0.0006; Table 4), indicating that the medium constituents significantly interacted with each other and had a significant impact on lipid accumulation. According to the ANOVA analysis, the linear terms of peptone, as well as the quadratic terms of starch, peptone, and inoculum size, were parameters that had a highly significant impact on lipid content of *F. soalni* (*p* < 0.01), but the remaining linear and interaction terms of the model showed no significance.

**Table 4:** ANOVA analysis of quadratic model for lipid content

Source	Coefficient estimate	Sum of Squares	Degrees of freedom	Mean Square	F-value	*p*-value
Model		479.03	9	53.27	33.19	0.0006
*A*-Starch	0.15	0.19	1	0.19	0.12	0.7436
*B*-Peptone	-1.66	22.04	1	22.04	13.73	0.009
*C*-Inoculum size	0.75	4.55	1	4.55	2.84	0.1530
*AB*	0.24	0.23	1	0.23	0.15	0.7190
*AC*	-0.22	0.20	1	0.20	0.12	0.7410
*BC*	1.10	4.84	1	4.84	3.01	0.1431
*A*²	10.40	399.18	1	399.18	248.72	< 0.0001
*B*²	3.45	43.83	1	43.83	27.31	0.0034
*C*²	3.43	43.42	1	43.42	27.06	0.0035
Residual		8.02	5	1.60		
Lack of Fit		6.85	3	2.28	3.90	0.2109
Pure Error		1.17	2	0.59		
Cor. Total		487.48	14			

Standard Deviation = 1.27, Mean = 12.94;,C.V.%= 9.78, R²= 0.984, Adjusted R²= 0.954, Predicted R²= 0.870, Adequate Precision = 15.07

A graph of the observed data versus expected results is shown in Fig. S3. It demonstrates that most data points were evenly spaced within a small region around the straight-line X = Y, indicating high model accuracy. As a result, this model could be exploited to guide the design space.

The second-order equation with coded parameters was expressed as follows (Eq. 4):

4 $$\begin{aligned}\mathrm{Y}_{\text{Lipid content}}&=3.73+0.15\:\mathrm{A}-1.66\:\mathrm{B}+0.75\:\mathrm{C}\\&+0.24\;\mathrm{AB}-0.22\:\mathrm{AC}+1.10\:\mathrm{BC}\\&+10.4\:\mathrm{A}^{2}+3.45\:\mathrm{B}^{2}+3.43\:\mathrm{C}^{2}\end{aligned}$$

**Fig. 4:**
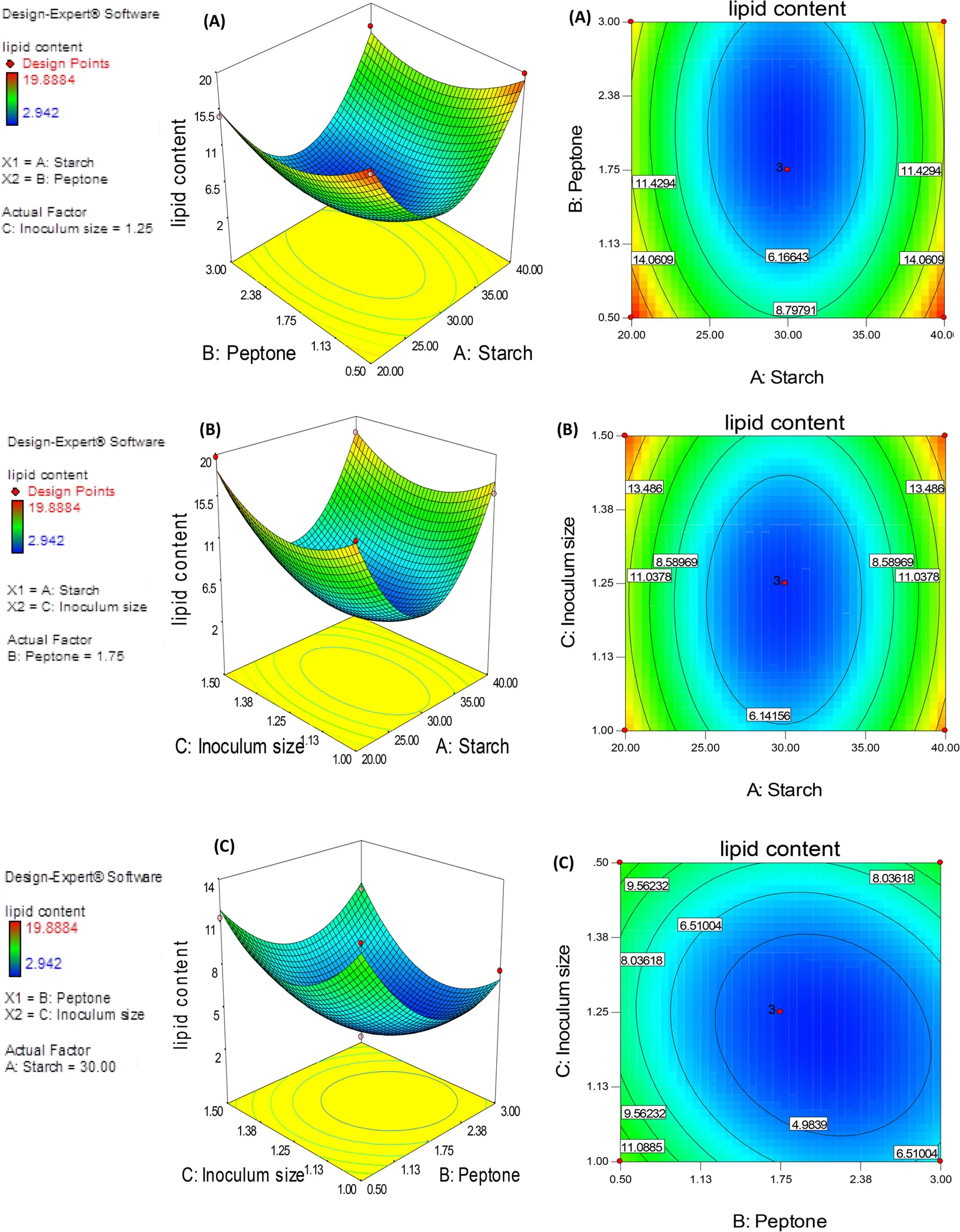
Three dimensional (3D) response surface and contour plots showing interactive effect of independent variables: (**A**) starch and peptone, (**B**) starch and inoculum size, and (**C**) peptone and inoculum size, on lipid content modeled by Box–Behnken design

Figure 4A illustrates the relationship between starch and peptone and its effect on lipid content of *F. solani* OQ275245. The findings indicate that the highest and lowest starch concentrations produced the highest lipid content. On the other hand, the negative value of the peptone estimate coefficient revealed that a significant increase in lipid content (14.06%) occurred when the peptone concentration was decreased to 0.5 g/L. These results are similar to those reported by Rasmey et al. [24], who observed that *Aspergillus caespitosus* produced more than 50% lipid when grown in a medium containing low nitrogen and high carbon. High carbon concentrations and limited nitrogen resources may improve the biosynthesis of triacylglycerol, which is subsequently stored in liposomes and improves lipid accumulation in oleaginous fungi [45]. Numerous studies also indicate that cell proliferation is a main process when the culture medium contains both nitrogen and carbon sources; but the accumulation of lipid often begins when the medium is carbon-loaded and nitrogen-deficient [46]. The activity of nicotinamide adenine dinucleotide isocitrate dehydrogenase decreases at low nitrogen medium concentrations. As a result, citrate builds up in the mitochondria and is subsequently transferred to the cytoplasm, where it is liberated by adenosine triphosphate citrate lyase to form acetyl-CoA, the building block of fatty acids [47]. Although nitrogen is a key factor in promoting lipid accumulation, its impact is reversed when lipid-producing strains accumulate lipids due to depletion of exogenous nitrogen in the growth medium [15]. Nitrogen limitation in the culture medium also stops cell proliferation, and cells store already produced lipids, which leads to lipid accumulation.

The change in lipid content of the *F. solani* strain depending on starch and inoculum size at a fixed peptone concentration of 1.75 g/l is shown in Fig. 4B. According to this figure, the highest lipid content in *F. solani* was recorded at the highest and lowest starch concentrations. However, as the inoculum size gradually increased, the lipid content also increased, reaching a maximum of 13.48% at 1.5%. Regarding this, Taskin et al. [48] found that *Yarrowia lipolytica* B9 had the highest biomass (4.38 g/l) and lipid content (28%) when cultivated in a medium containing 3% of the inoculum concentration. Chen et al. [49] shown in another investigation that *Cutaneotrichosporon cutaneum* (syn., *Trichosporon cutaneum*) cultivated on corncob acid hydrolysate produced the highest biomass and lipids when the inoculum size was 5%. According to Abdelhamid et al. [50], *Penicillium commune* NRC2016 produced the maximum lipids (30.37%) when the inoculum size was 0.75%, while the lowest value (28.50%) was achieved when the inoculum size was 0.5%.

Three-dimensional graphs and contour plots as a function of peptone and inoculum size are presented in Fig. 4C. The figure indicates that the lipid content of *F. solani* OQ275245 is significantly impacted by changes in peptone and that it increases with decreasing peptone concentration. On the other hand, lipid content increased non-significantly (*p* ˃ 0.05) with increasing inoculum concentration. Under unfavorable environmental circumstances and nutrient deprivations, the accumulation of cellular lipids by lipid-producing strains typically began after cell development has stopped. This was particularly evident in cases of nitrogen deprivation, where the *Fusarium* isolate significantly enhanced lipid accumulation with less nitrogen supply, while inhibiting or decreasing the growth of the fungal strain, as mentioned previously [27, 51].

### Optimization and verification

**Fig. 5:**
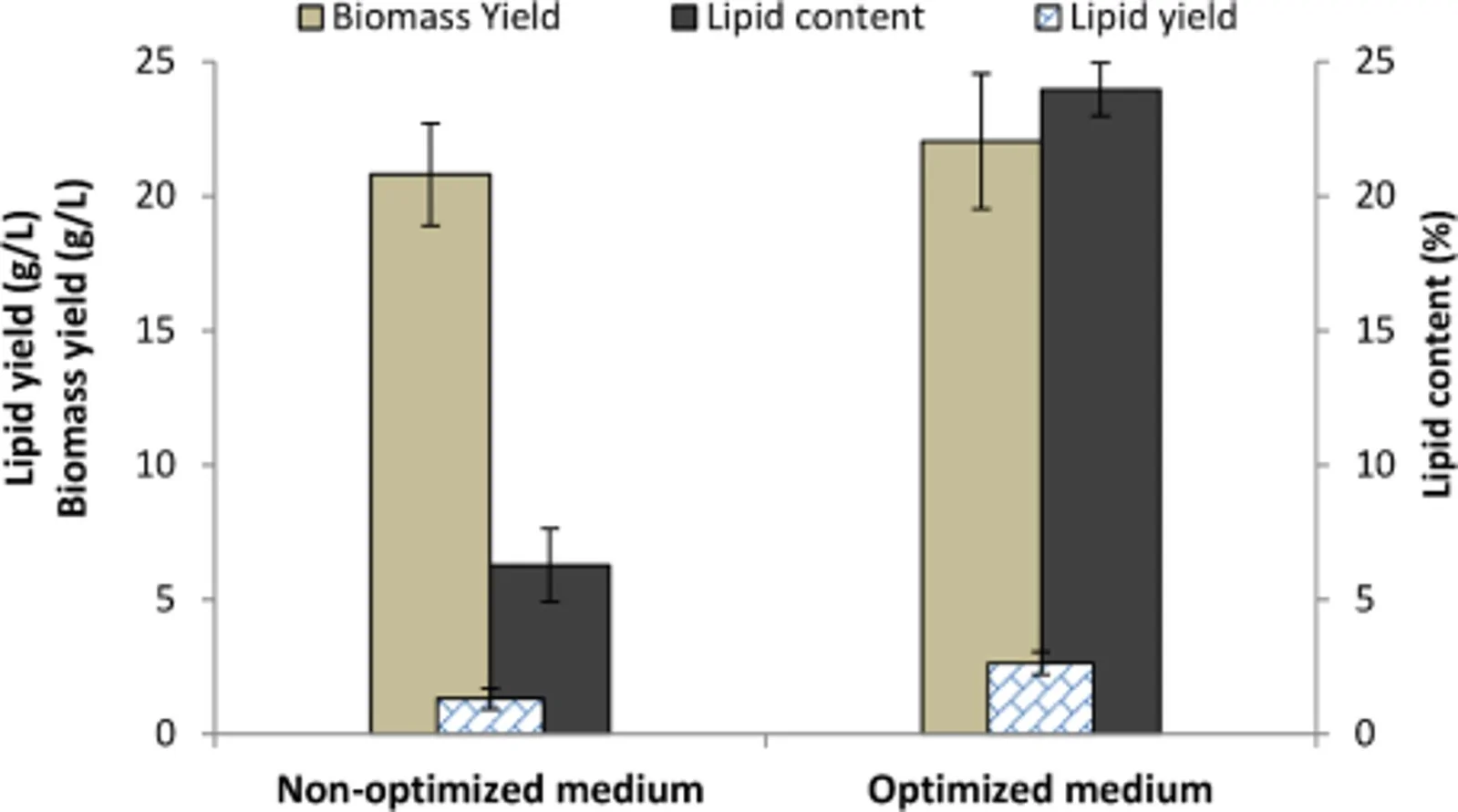
Biomass yield, lipid yield and lipid content of *F. solani* OQ275245 before and after optimization by Box-Behnken Design

**Fig. 6:**
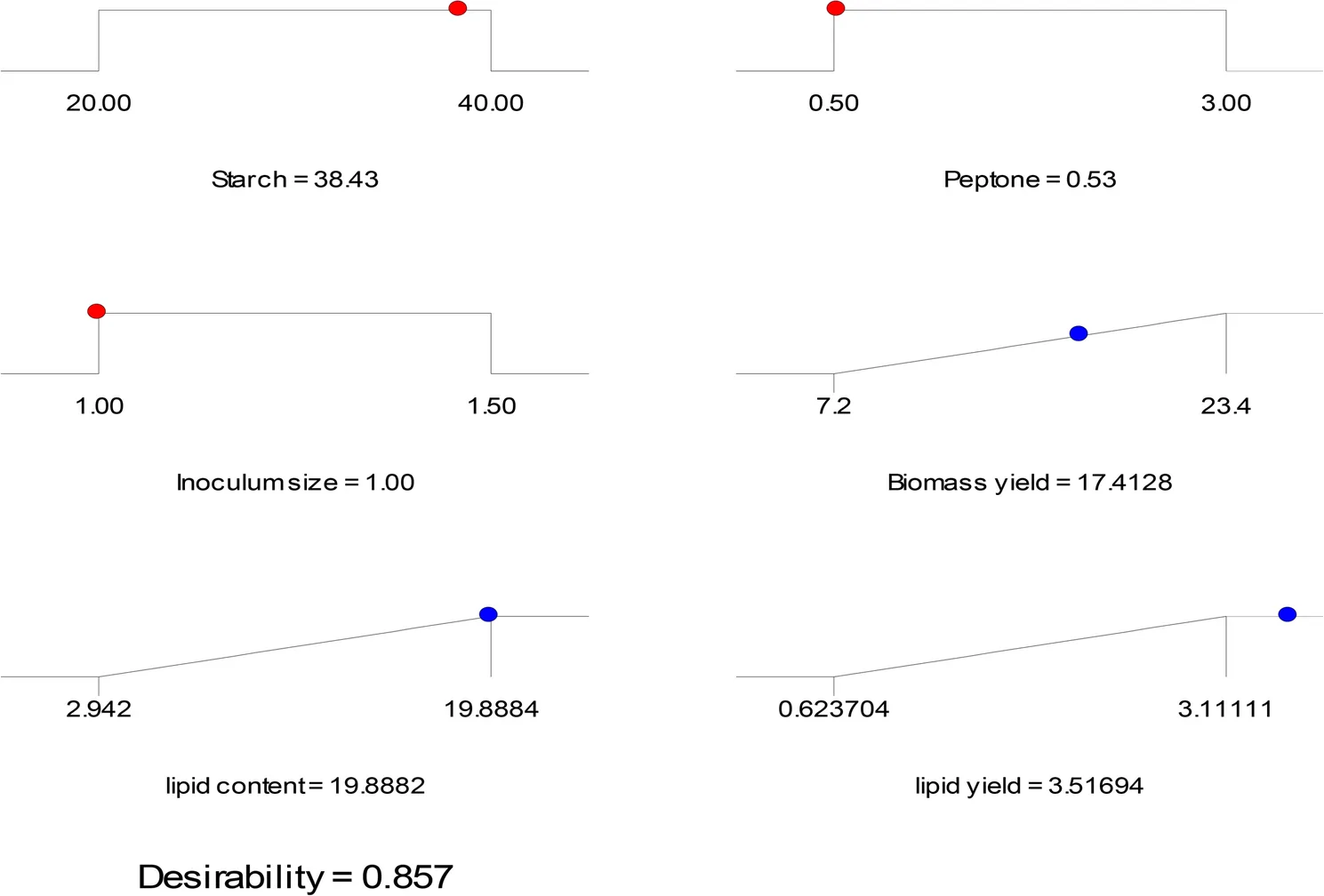
Desirability of the optimized culture media model based on Box-Behnken Design

Three additional experiments were conducted in shake flasks under optimal conditions obtained by BBD (starch 38.43 g/L, peptone 0.53 g/L, and inoculum size 1.0%; Fig. 5) to validate the optimized growth conditions. Figure 5 displays data on biomass, lipid yield, and lipid content for *F. solani* OQ275245 before and after optimization. Under optimized culture conditions, biomass, lipid yield, and lipid content were 22.04 g/L ± 2.51, 5.28 ± 0.55 g/L, and 23.96 ± 0.99%, respectively. These values are higher than those for the original medium (20.80 ± 1.90, 1.31 ± 0.38 g/L, and 6.29 ± 1.36%, respectively; Fig. 5) and approximately close to the expected values (17.41 g/L, 3.52 g/L, and 19.89%, respectively; Fig. 6). It was shown that under optimal conditions, fungal biomass yield, lipid yield and lipid content increased 1.06-, 4.03-, and 3.81-fold, respectively. This may help minimize production costs, as peptone and starch concentrations were lower in the optimized growth medium than in the original non-optimized medium. The above findings show that the expected and observed results coincide well and also imply that the models for biomass yield, lipid yield, and lipid content are accurate and reasonable. *Fusarium solani* OQ275245 strain produced higher biomass and lipid production compared to *Aspergillus terreus* Mekky221 grown under optimal growth circumstances for ten days [52]. Moreover, the optimized lipid yield produced by the studied fungal strain is similar to that obtained in the study by Thangavelu et al. [51], in which *Candida tropicalis* ASY2 was grown under optimized conditions with a bioreactor airflow rate of 5.992 L/min, yeast extract of 0.5 g/L, and starch concentration of 15.33 g/L. This proves that *F. solani* OQ275245 is an oil-producing microorganism that can potentially be employed in the industrial production of microbial oils.

### Composition of fatty acid methyl esters and biodiesel characteristics of lipid

To assess the feasibility of employing this oleaginous fungus as a biodiesel feedstock, the fatty acid composition of *F. solani* OQ275245 was evaluated by analyzing fatty acid methyl esters (FAMEs) generated from transesterified fungal lipids. The quality and quantity of lipids and their fatty acid profiles affect the quality of the produced biodiesel.

Table 5 shows the fatty acid composition of lipids produced by the *F. solani* strain. It shows the presence of high amounts of long chain saturated fatty acids, including myristic acid (C14:0; 45.78%), palmitic acid (C16:0; 42.22%), and stearic acid (C18:0; 6.23). Low proportions of other fatty acids were found, including oleic acid (C18:1; 1.57%), and linoleic acid (C18:2; 4.01%). The current findings were consistent with the results of the study by Al-Zaban and Abd El-Aziz [53] who stated that *Aspergillus terreus* KC462061 had a fatty acid composition of C14:0, C16:0, C18:0, C18:1 and C18:2, however, the amount of saturated (SFA) and unsaturated fatty acids (USFA) was varied. Additionally, *Rhizopus stolonifer* grown on flax straw produced a fatty acid profile comparable to that of the *F. solani* strain, with palmitic acid constituting 42.18% of the total lipid [54]. Another research on the oleaginous yeast *Rhodosporidium toruloides* 21167 cultivated on hydrolysate of cassava starch extract revealed that it contained mainly myristic acid, palmitic acid, stearic acid, oleic acid, and linoleic acid [55]. Palmitic acid, stearic acid, and oleic acid are considered possible components of interest because of their higher-value lipids characteristics, enhanced oxidative stability, and adaptability in biodiesel production [56].

**Table 5:** GC/MS analysis of fatty acid profile

Fatty acids	Lipid numbers	% content of FA in F. solani OQ275245
Myristic acid	C14:0	45.78
Palmitic acid	C16:0	42.22
Stearic acid	C18:0	6.23
Oleic acid	C18:1	1.57
Linoleic acid	C18:2	4.01
Total		100
Saturated fatty acid		94.2
Unsaturated fatty acid		5.6
- Monounsaturated (MUFA)		1.6
- Polyunsaturated (PUFA)		4.0
Total		100

The quality of biodiesel depends on the fatty acid profile, which should contain higher percentages of saturated, and monounsaturated fatty acids and lower proportions of polyunsaturated fatty acids [57].

According to the current study, the fatty acid profile of *F. solani* included a high level of SFA (94.2%) and a low percentage of MUFA and PUFA (5.6%; Table 5). The high saturated fatty acid content of the tested fungal strain provides it with an advantage that makes it highly suitable for the production of biodiesel with high fuel effectiveness. These advantages include low levels of nitrogen oxide and increased oxidative stability and cetane number. Conversely, high levels of unsaturated fatty acids exhibit poor oxidative stability, particularly after exposure to air and/or light for extended periods and storage above room temperature [58]. These results are consistent with earlier results for biodiesel obtained from the lipids of oleaginous microalgae and fungi, which tended to be more saturated and provided more advantageous biodiesel qualities [59, 60].

Various physicochemical characteristics of biodiesel obtained from *F. solani* OQ275245 were identified in order to assess its viability as an alternative to diesel fuel. Table 6 compares the properties of biodiesel produced from fungal isolate with conventional diesel fuel [61, 62], the American biodiesel standard ASTM D6751, and the European biodiesel standard EN14214. The most important characteristic of diesel fuel that influences emissions and engine performance is the cetane number (CN). In this study, the CN of biodiesel from *F. solani* (69.77) was greater than that of conventional diesel fuel and the lowest amount suggested by European and American standard limits. Biodiesel produced from *F. solani* may have a higher CN due to its high saturated fatty acid content. Fuels with a higher CN may improve engine performance and acceleration [63]. Another property is the saponification value (SV), which defined as the number of grams of sodium hydroxide needed to fully saponify all triglyceride esters in one gram of specific oil [64]. The SV recorded for this biodiesel was 215.46.9 mg KOH/g, exceeding the value of 192.70 mg KOH/g stated by Ibrahim et al. [9].

**Table 6:** Fuel characteristics of *F. solani* OQ275245 biodiesel, ASTM D6751-08, EN 14,214, and conventional diesel

Biodiesel from F. solani	CN	SV	IV	OS	DU	ρ	kυ	HHV	CFPP	CP	PP	LCSF
	69.77	215.46	8.26	7.40	9.60	1.10	1.33	35.56	11.29	17.22	11.87	8.84
US biodiesel standard ASTM D6751-08	≥ 47	NA	NA	≥ 3	NA	0.86–0.9	1.9-6.0	35	-13-5	-3-12	NA	NA
EU Standard biodiesel EN14214	≥ 51	NA	≤ 120	≥ 8	NA	0.87	3.5-5.0	42	-20-5	˃4	NA	NA
Diesel [61, 62]	45–55	NA	6	NA	NA	0.845	2.91-4	42-45.5	NA	NA	-20	NA

*CN* Cetane Number, *SV* Saponification Value (mg KOH g^–1^), *IV* Iodine value (gI_2_100 g^–1^ oil), *OS* Oxidation Stability (h), *DU* Degree of Unsaturation (%), *ρ* Density (g cm^–3^), *kυ* kinematic viscosity (mm^2^ s^–1^), *HHV* High Heating Value (MJ kg^–1^), CFPP Cold Filter Plugging Point (°C), *CP* Cloud Point (°C), *PP* Pour Point (°C), *LCSF* Long Chain Saturated Factor (%), *NA* not available

The degree of unsaturation of the oil is measured by the iodine value (IV), which is a key characteristic of biodiesel fuel. A high iodine value is always achieved with a high degree of unsaturation and high vulnerability to oxidation, while a low iodine value provides suitable oxidation stability [65]. According to the current results, the *Fusarium* strain meets international biodiesel standards due to its low IV (8.26 gI_2_100/g oil) and high oxidative stability (OS; 7.40 h). This demonstrates the stability of the biodiesel produced from the *Fusarium* lipid. Biodiesel produced by the *F. solani* strain had a low degree of unsaturation (UD; 9.6%), due to the decrease in the content of unsaturated fatty acids. For commercial biodiesel, a lower value of UD is an excellent indicator.

The density (*p*) of *Fusarium* lipids was 1.1 g/cm^3^ (Table 6), which is relatively higher than the limits of American and European standards. Fuel injection performance is directly dependent on density, which increases as chain length decreases and the number of double bonds increases. According to kinematic viscosity (υ) results, *Fusarium* biodiesel (1.33 mm^2^/s) has a lower value than both conventional diesel fuel and biodiesel according to ASTM D6751-08 and EN14214 standards. Density and kinematic viscosity affect combustion, engine performance, and exhaust emissions [66].

The fuel’s heating value shows the amount of energy released during combustion of a unit of fuel. Fungal biodiesel has a high heating value (35.56 MJ/kg), which is close to the limits of American standards. Cold filter plugging point (CFPP), cloud point (CP), pour point (PP), the amount of long-chain SFAs and USFAs are used to calculate the fuel’s stability at low temperatures. Biodiesel fuel can block filters or limit flow during ignition at low temperature, because of crystallization by excess SFAs. In this study, a high increase in saturated fatty acids in the *Fusarium* lipid led to an increase in CFPP (11.29 °C), CP (17.22 °C), and PP (11.87 °C). When one or more unsaturated fatty acids are present alongside saturated fatty acids such as C16 and C18, the fuel’s propensity to solidify at low temperatures is decreased, biodiesel’s viscosity is also decreased, and the blockage point characteristics between the filters are enhanced [67]. Nevertheless, the biofuel will have poor oxidative stability if there is an excess of USFA, particularly PUFA [68]. Furthermore, the LCSF of the *Fusarium* fuel was 8.84% (Table 6), which was almost similar to the 7.48% recorded by Fawzy et al. [63]. These findings validate the occurrence of long chain SFAs in high amounts with low USFA levels in the fungal strain and demonstrate that biodiesel generated by *F. solani* OQ275245 is appropriate for use in both hot and cold temperature areas.

It is clear that the biodiesel fuel qualities of *F. solani* comply with most requirements reported in both international standards and conventional diesel. Hence, the new, indigenous strain *F. solani* OQ275245 represents an attractive and potential alternative for the production of biodiesel.

## Conclusions

Biodiesel produced from filamentous fungi is considered one of the most promising renewable biofuels. To support the viability of biofuels as energy substitutes for fossil fuels, it is crucial to improve the productivity of biomass and lipids in fungal cultures. In this investigation, an indigenous isolate of F. solani OQ275245 was assessed for its ability to produce microbial lipids and its potential use as a laboratory-scale biodiesel feedstock. The RSM and BBD methodologies successfully optimized selected components of the culture medium (starch, peptone, and inoculum concentration), resulting in enhanced biomass production, lipid yield, and lipid content compared with the non-optimized medium. Under the optimized conditions (38.43 g/L starch, 0.53 g/L peptone, and 1.0% inoculum size), the fungal strain produced 22.04 ± 2.51 g/L biomass, 5.28 ± 0.55 g/L lipid yield, and 23.96 ± 0.99% lipid content. Saturated fatty acids such as myristic acid, palmitic acid and stearic acid were predominant in the fatty acid profile of the fungal lipids, and the properties of the biodiesel were generally in line with international biodiesel criteria. These results suggest that F. solani OQ275245 may represent a promising source of microbial lipid for laboratory-scale biodiesel production. However, several limitations should be considered. The study was performed using a single fungal strain under shake-flask conditions, and only selected nutritional parameters were optimized, while other operational and environmental conditions, such as different carbon and nitrogen sources, pH, and temperature, were not evaluated. Furthermore, fungal identification was based on 28 S rRNA sequence analysis, whereas multilocus or genome-based characterization was beyond the scope of the current study. The biosafety aspects, potential pathogenicity, and economic feasibility of large-scale cultivation were also not examined. Therefore, further study is needed, including comprehensive strain characterization, utilization of low-cost substrates, biosafety evaluation, economic feasibility, process scale-up, and experimental validation of fuel performance, to evaluate the potential use of Fusarium solani OQ275245 in industrial applications.

## Supplementary Information

Supplementary Material 1.

## Data Availability

The sequence generated in this study has been deposited in the GenBank database under accession number OQ275245.

## Abbreviations

RSM: Response surface methodology
BBD: Box–Behnken design
ANOVA: Analysis of variance
C14: 0:Myristic acid
C16: 0:Palmitic acid
C18: 0:Stearic acid
C18: 1:Oleic acid
C18: 2:Linoleic acid
*CN*: Cetane number
*SV*: Saponification Value (mg KOH g^–1^)
*IV*: Iodine value (gI_2_100 g^–1^ oil)
*OS*: Oxidation Stability (h)
*DU*: Degree of Unsaturation (%)
*ρ*: Density (g cm^–3^)
*kυ*: kinematic viscosity (mm^2^ s^–1^)
*HHV*: High Heating Value (MJ kg^–1^)
CFPP: Cold Filter Plugging Point (°C),
*CP*: Cloud Point (°C),
*PP*: Pour Point (°C),
LCSF: Long Chain Saturated Factor (%),
