# Supplementary material for: Improving growth and lipid accumulation in a novel oleaginous fungus Fusarium solani as a promising feedstock for biodiesel: optimization of growth medium and fatty acid characterization
Source: BMC Microbiol. 2026 Jul 21;26:835. doi: 10.1186/s12866-026-05396-8 (PMC13602762; doi:10.1186/s12866-026-05396-8)
Supplement: Supplementary file 1 [file 12866_2026_5396_MOESM1_ESM.docx]

**Supplementary data**

Improving growth and lipid accumulation in a novel oleaginous fungus *Fusarium solani* as a promising feedstock for biodiesel: Optimization of growth medium and fatty acid characterization

Sedky H. A. Hassan^1^, Mustafa A. Fawzy^2,3^*, Hana Sonbol^4^, Albandary N. Alsaloom^4^, Mostafa Koutb^2,5^, and Amany G. Ibrahim^3,6^*

^1^	Department of Biology, College of Science, Sultan Qaboos University, Muscat 123, Oman

^2^	Botany and Microbiology Department, Faculty of Science, Assiut University, Assiut 71516, Egypt

^3^	Biology Department, College of Sciences, Taif University, P.O. Box 11099, Taif 21944, Saudi Arabia

^4^	Department of Biology, College of Science, Princess Nourah bint Abdulrahman University, P.O. Box 84428, Riyadh 11671, Saudi Arabia

^5^	Department of Biology, Faculty of Science, Umm Al-Qura University, Makkah 24381, Saudi Arabia;

^6^	Botany Department, Faculty of Women for Arts, Science and Education, Ain Shams University,
Cairo 11566, Egypt

*****	Correspondence: [mustafa.fawzy@aun.edu.eg](mailto:mustafa.fawzy@aun.edu.eg); Tel: +201002931424; Amany.gomaa@women.asu.edu.eg; Tel: +201020526488

**Fig. S1.**  Actual against predicted values plot for biomass yield of *F. solani*.

**Fig. S2.** Actual against predicted values plot for lipid yield by F*. solani.*

**Fig. S3.** Actual against predicted values plot for lipid content of *F. solani*.
